# 4-Cyano-1-methyl­pyridinium bromide

**DOI:** 10.1107/S1600536812030449

**Published:** 2012-07-10

**Authors:** Michael N. Kammer, Joel T. Mague, Lynn V. Koplitz

**Affiliations:** a Department of Physics, Loyola University, New Orleans, LA 70118, USA; b Department of Chemistry, Tulane University, New Orleans, LA 70118, USA; c Department of Chemistry, Loyola University, New Orleans, LA 70118, USA

## Abstract

In the crystal of the title mol­ecular salt, C_7_H_7_N_2_^+^·Br^−^, the cations form inversion dimers *via* weak pairwise C—H⋯N hydrogen bonds; their mean planes are separated by 0.292 (6) Å. Weak C—H⋯Br inter­actions involving all of the remaining H atoms tie the cations and anions together into sets of inter­penetrating sheets. The title compound is isostructural with its iodide analogue.

## Related literature

For the structure of the 4-cyano-1-methyl­pyridinium iodide salt, see: Kammer *et al.* (2012 ▶). For the structure of 3-cyano-1-methyl­pyridinium bromide, see: Mague *et al.* (2005 ▶). For the structure of 3-cyano-1-methyl­pyridinium chloride, see: Koplitz *et al.* (2003 ▶.
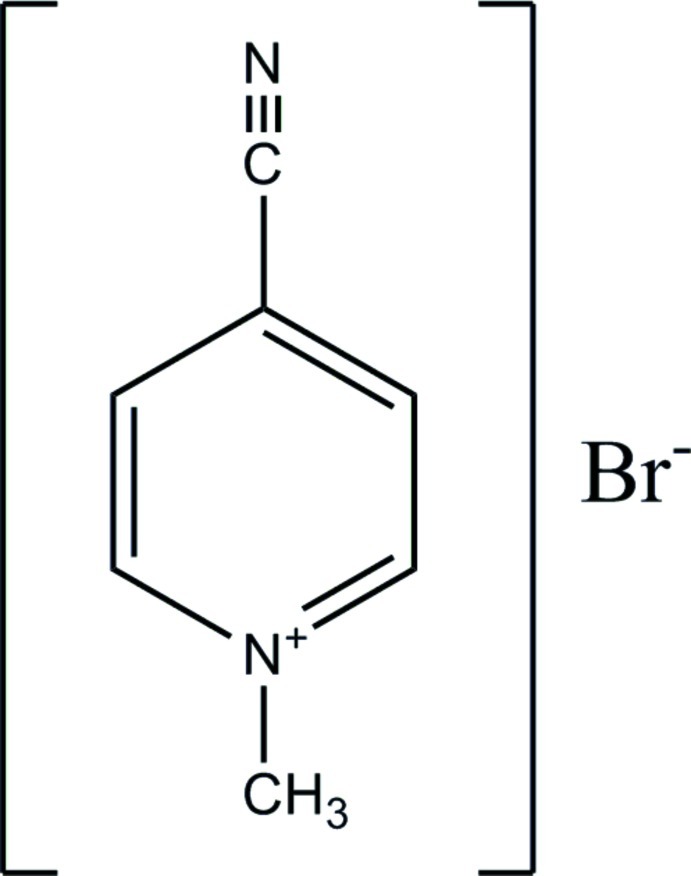


## Experimental

### 

#### Crystal data

C_7_H_7_N_2_^+^·Br^−^
*M* *_r_* = 199.06
Monoclinic, 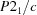

*a* = 4.5447 (16) Å
*b* = 11.285 (4) Å
*c* = 15.551 (6) Å
β = 96.455 (5)°
*V* = 792.5 (5) Å^3^
*Z* = 4
Mo *K*α radiation
μ = 5.11 mm^−1^
*T* = 100 K
0.26 × 0.22 × 0.13 mm

#### Data collection

Bruker SMART APEX CCD diffractometer
Absorption correction: numerical (*SADABS*; Bruker, 2009 ▶) *T*_min_ = 0.351, *T*_max_ = 0.563
12985 measured reflections
2050 independent reflections
1864 reflections with *I* > 2σ(*I*)
*R*_int_ = 0.065

#### Refinement

*R*[*F* ^2^ > 2σ(*F* ^2^)] = 0.027
*wR*(*F* ^2^) = 0.073
*S* = 1.07
2050 reflections
92 parameters
H-atom parameters constrained
Δρ_max_ = 0.42 e Å^−3^
Δρ_min_ = −0.72 e Å^−3^

### 

Data collection: *APEX2* (Bruker, 2010 ▶); cell refinement: *SAINT* (Bruker, 2009 ▶); data reduction: *SAINT*; program(s) used to solve structure: *SHELXS97* (Sheldrick, 2008 ▶); program(s) used to refine structure: *SHELXL97* (Sheldrick, 2008 ▶); molecular graphics: *SHELXTL* (Sheldrick, 2008 ▶); software used to prepare material for publication: *SHELXTL*.

## Supplementary Material

Crystal structure: contains datablock(s) I, global. DOI: 10.1107/S1600536812030449/hb6887sup1.cif

Structure factors: contains datablock(s) I. DOI: 10.1107/S1600536812030449/hb6887Isup2.hkl

Supplementary material file. DOI: 10.1107/S1600536812030449/hb6887Isup3.cml

Additional supplementary materials:  crystallographic information; 3D view; checkCIF report

## Figures and Tables

**Table 1:** Hydrogen-bond geometry (Å, °)

*D*—H⋯*A*	*D*—H	H⋯*A*	*D*⋯*A*	*D*—H⋯*A*
C3—H3⋯N2^i^	0.95	2.48	3.357 (2)	153
C5—H5⋯Br1^ii^	0.95	2.72	3.626 (2)	160
C6—H6⋯Br1^iii^	0.95	2.78	3.6779 (19)	157
C1—H1*B*⋯Br1^iv^	0.98	2.89	3.735 (2)	144
C1—H1*C*⋯Br1^iii^	0.98	2.82	3.755 (2)	160
C2—H2⋯Br1^v^	0.95	2.79	3.6253 (18)	147

Symmetry codes: (i) 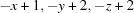; (ii) 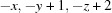; (iii) 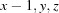; (iv) 
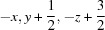
; (v) 
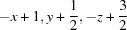
.

**Fig. 1.**
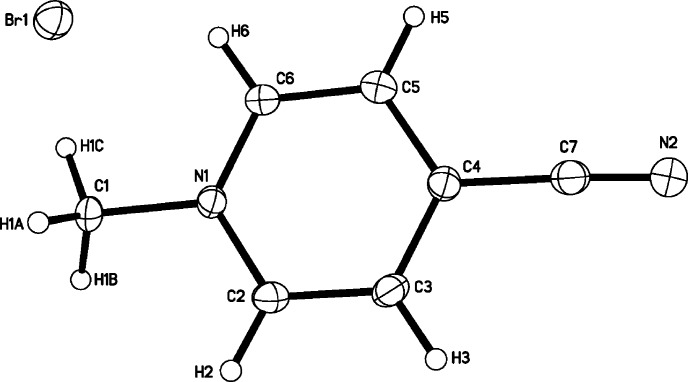
Perspective view of the asymmetric unit. Displacement ellipsoids are drawn at the 50% probability level.

**Fig. 2.**
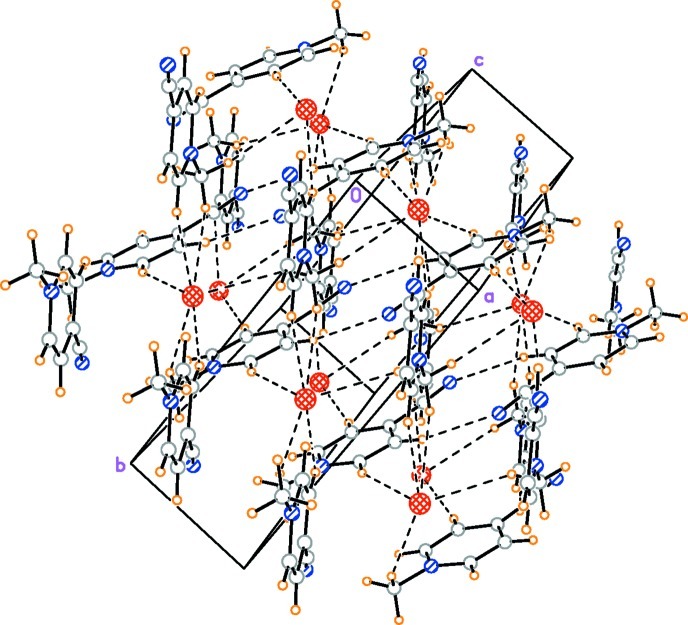
Packing of the title compound showing the interpenetrating sheet structure. Color key: C = gray, H = orange, Br = red, N = blue.

## supplementary crystallographic information

### Comment

In the title compound the cations form dimers *via* weak, pairwise C2—H2···N2 hydrogen bonds. In these cations the six-membered rings are parallel within 0.10 ° with the mean planes separated by 0.292 (6) Å. The remaining hydrogen atoms form weak interactions with five neighboring bromide ions (Table 1) to generate a three dimensional network of interpenetrating planes (Fig. 2).

4-Cyano-1-methylpyridinium bromide is isostructural with the corresponding iodide (Kammer *et al.*, 2012). By contrast, the structure of the bromide salt of the isomeric 3-cyano-1-methylpyridinium cation differs markedly from that of its iodide salt but is isostructural with its chloride salt (Koplitz *et al.*, 2003; Mague *et al.*, 2005). Also, in the title compound each anion participates in five C—H···Br contacts in the range 2.27–2.89 Å with a sixth, essentially van der Waals contact of 3.03 Å. This differs markedly from the isomeric 3-cyano-1-methylpyridinium bromide (Mague *et al.*, 2005) where each bromide ion is contacted by four C—H groups all in the same plane.

### Experimental

4-Cyanopyridine (10.55 g) was dissolved in benzene (40 ml). Iodomethane (9.5 ml) was added to this solution slowly with stirring and the solution was refluxed for 75 minutes. Yellow solid 4-cyano-*N*-methylpyridinium iodide (m.p. 189–193° C) was collected by vacuum filtration. An aqueous solution of this iodide salt was passed down a column of polymer-supported bromide ion-exchange resin (Aldrich calalogue No. 51,376–8) and the eluate evaporated to dryness. Yellow slabs for the structure determination were grown by slow evaporation of a solution of the compound in a 1:1 (*v*/*v*) mixture of acetonitrile and ethanol under ambient conditions (m.p. 213° C).

### Refinement

H-atoms attached to carbon were placed in calculated positions (C—H = 0.95 - 0.98 Å) and included as riding contributions with isotropic displacement parameters 1.2 - 1.5 times those of the attached carbon atoms.

### Crystal data

**Table:**

C_7_H_7_N_2_^+^·Br^−^	*F*(000) = 392
*M**_r_* = 199.06	*D*_x_ = 1.668 Mg m^−^^3^
Monoclinic, *P*2_1_/*c*	Mo *K*α radiation, λ = 0.71073 Å
Hall symbol: -P 2ybc	Cell parameters from 9556 reflections
*a* = 4.5447 (16) Å	θ = 2.6–29.1°
*b* = 11.285 (4) Å	µ = 5.11 mm^−^^1^
*c* = 15.551 (6) Å	*T* = 100 K
β = 96.455 (5)°	Slab, yellow
*V* = 792.5 (5) Å^3^	0.26 × 0.22 × 0.13 mm
*Z* = 4	

### Data collection

**Table:**

Bruker SMART APEX CCD diffractometer	2050 independent reflections
Radiation source: fine-focus sealed tube	1864 reflections with *I* > 2σ(*I*)
Graphite monochromator	*R*_int_ = 0.065
φ and ω scans	θ_max_ = 29.1°, θ_min_ = 2.2°
Absorption correction: numerical (*SADABS*; Bruker, 2009)	*h* = −6→6
*T*_min_ = 0.351, *T*_max_ = 0.563	*k* = −15→15
12985 measured reflections	*l* = −20→20

### Refinement

**Table:**

Refinement on *F*^2^	Primary atom site location: structure-invariant direct methods
Least-squares matrix: full	Secondary atom site location: difference Fourier map
*R*[*F*^2^ > 2σ(*F*^2^)] = 0.027	Hydrogen site location: inferred from neighbouring sites
*wR*(*F*^2^) = 0.073	H-atom parameters constrained
*S* = 1.07	*w* = 1/[σ^2^(*F*_o_^2^) + (0.039*P*)^2^ + 0.1785*P*] where *P* = (*F*_o_^2^ + 2*F*_c_^2^)/3
2050 reflections	(Δ/σ)_max_ = 0.002
92 parameters	Δρ_max_ = 0.42 e Å^−^^3^
0 restraints	Δρ_min_ = −0.72 e Å^−^^3^

### Special details

**Table:**

Experimental. The diffraction data were obtained from 3 sets of 400 frames, each of width 0.5 °. in omega, colllected at phi = 0.00, 90.00 and 180.00 °. and 2 sets of 800 frames, each of width 0.45 ° in phi, collected at omega = -30.00 and 210.00 °. The scan time was 20sec/frame.
Geometry. All e.s.d.'s (except the e.s.d. in the dihedral angle between two l.s. planes) are estimated using the full covariance matrix. The cell e.s.d.'s are taken into account individually in the estimation of e.s.d.'s in distances, angles and torsion angles; correlations between e.s.d.'s in cell parameters are only used when they are defined by crystal symmetry. An approximate (isotropic) treatment of cell e.s.d.'s is used for estimating e.s.d.'s involving l.s. planes.
Refinement. Refinement of *F*^2^ against ALL reflections. The weighted *R*-factor *wR* and goodness of fit *S* are based on *F*^2^, conventional *R*-factors *R* are based on *F*, with *F* set to zero for negative *F*^2^. The threshold expression of *F*^2^ > σ(*F*^2^) is used only for calculating *R*-factors(gt) *etc*. and is not relevant to the choice of reflections for refinement. *R*-factors based on *F*^2^ are statistically about twice as large as those based on *F*, and *R*- factors based on ALL data will be even larger. H-atoms were placed in calculated positions (C—H = 0.95 - 0.98 Å) and included as riding contributions with isotropic displacement parameters 1.2 - 1.5 times those of the attached carbon atoms.

### Fractional atomic coordinates and isotropic or equivalent isotropic displacement parameters (Å^2^)

**Table:**

	*x*	*y*	*z*	*U*_iso_*/*U*_eq_	
Br1	0.38940 (3)	0.377230 (14)	0.850526 (11)	0.01629 (9)	
N1	−0.0140 (3)	0.65445 (13)	0.81550 (10)	0.0142 (3)	
N2	0.3192 (4)	0.93928 (14)	1.08002 (11)	0.0246 (4)	
C1	−0.1282 (4)	0.58354 (16)	0.73879 (11)	0.0181 (3)	
H1A	0.0322	0.5352	0.7202	0.027*	
H1B	−0.2044	0.6368	0.6917	0.027*	
H1C	−0.2881	0.5318	0.7537	0.027*	
C2	0.1619 (4)	0.74794 (15)	0.80376 (11)	0.0166 (3)	
H2	0.2143	0.7655	0.7477	0.020*	
C3	0.2658 (4)	0.81810 (15)	0.87348 (12)	0.0180 (3)	
H3	0.3935	0.8832	0.8663	0.022*	
C4	0.1801 (4)	0.79167 (15)	0.95433 (11)	0.0160 (3)	
C5	0.0060 (4)	0.69212 (15)	0.96585 (12)	0.0184 (3)	
H5	−0.0465	0.6717	1.0214	0.022*	
C6	−0.0873 (4)	0.62429 (14)	0.89416 (13)	0.0172 (4)	
H6	−0.2043	0.5557	0.9003	0.021*	
C7	0.2641 (5)	0.87129 (15)	1.02619 (14)	0.0202 (4)	

### Atomic displacement parameters (Å^2^)

**Table:**

	*U*^11^	*U*^22^	*U*^33^	*U*^12^	*U*^13^	*U*^23^
Br1	0.01641 (13)	0.01499 (12)	0.01814 (14)	−0.00147 (5)	0.00490 (8)	−0.00055 (5)
N1	0.0167 (7)	0.0123 (6)	0.0134 (7)	0.0003 (5)	0.0003 (5)	−0.0007 (5)
N2	0.0304 (9)	0.0227 (8)	0.0205 (8)	−0.0056 (6)	0.0020 (7)	−0.0010 (6)
C1	0.0250 (9)	0.0148 (8)	0.0142 (8)	−0.0032 (6)	0.0000 (7)	−0.0033 (7)
C2	0.0178 (8)	0.0160 (7)	0.0161 (8)	−0.0006 (6)	0.0021 (6)	0.0031 (6)
C3	0.0193 (8)	0.0144 (7)	0.0198 (9)	−0.0036 (6)	0.0002 (6)	0.0025 (7)
C4	0.0172 (8)	0.0148 (7)	0.0156 (8)	0.0009 (6)	−0.0006 (6)	0.0004 (6)
C5	0.0207 (8)	0.0188 (8)	0.0161 (8)	−0.0014 (6)	0.0034 (6)	0.0018 (7)
C6	0.0203 (9)	0.0157 (8)	0.0156 (9)	−0.0020 (6)	0.0023 (7)	0.0018 (6)
C7	0.0209 (10)	0.0200 (9)	0.0196 (10)	−0.0022 (6)	0.0018 (8)	0.0030 (7)

### Geometric parameters (Å, º)

**Table:**

N1—C6	1.347 (2)	C2—H2	0.9500
N1—C2	1.349 (2)	C3—C4	1.390 (2)
N1—C1	1.481 (2)	C3—H3	0.9500
N2—C7	1.142 (3)	C4—C5	1.397 (2)
C1—H1A	0.9800	C4—C7	1.451 (3)
C1—H1B	0.9800	C5—C6	1.379 (3)
C1—H1C	0.9800	C5—H5	0.9500
C2—C3	1.382 (2)	C6—H6	0.9500
			
C6—N1—C2	122.10 (15)	C2—C3—H3	120.6
C6—N1—C1	119.66 (14)	C4—C3—H3	120.6
C2—N1—C1	118.24 (14)	C3—C4—C5	120.59 (16)
N1—C1—H1A	109.5	C3—C4—C7	119.17 (16)
N1—C1—H1B	109.5	C5—C4—C7	120.19 (16)
H1A—C1—H1B	109.5	C6—C5—C4	118.02 (16)
N1—C1—H1C	109.5	C6—C5—H5	121.0
H1A—C1—H1C	109.5	C4—C5—H5	121.0
H1B—C1—H1C	109.5	N1—C6—C5	120.58 (15)
N1—C2—C3	119.84 (16)	N1—C6—H6	119.7
N1—C2—H2	120.1	C5—C6—H6	119.7
C3—C2—H2	120.1	N2—C7—C4	175.7 (2)
C2—C3—C4	118.74 (16)		
			
C6—N1—C2—C3	−1.9 (2)	C3—C4—C5—C6	−2.6 (2)
C1—N1—C2—C3	178.19 (16)	C7—C4—C5—C6	175.03 (17)
N1—C2—C3—C4	−1.4 (2)	C2—N1—C6—C5	2.9 (3)
C2—C3—C4—C5	3.6 (2)	C1—N1—C6—C5	−177.16 (16)
C2—C3—C4—C7	−174.06 (16)	C4—C5—C6—N1	−0.6 (3)

### Hydrogen-bond geometry (Å, º)

**Table:**

*D*—H···*A*	*D*—H	H···*A*	*D*···*A*	*D*—H···*A*
C3—H3···N2^i^	0.95	2.48	3.357 (2)	153
C5—H5···Br1^ii^	0.95	2.72	3.626 (2)	160
C6—H6···Br1^iii^	0.95	2.78	3.6779 (19)	157
C1—H1*B*···Br1^iv^	0.98	2.89	3.735 (2)	144
C1—H1*C*···Br1^iii^	0.98	2.82	3.755 (2)	160
C2—H2···Br1^v^	0.95	2.79	3.6253 (18)	147

Symmetry codes: (i) −*x*+1, −*y*+2, −*z*+2; (ii) −*x*, −*y*+1, −*z*+2; (iii) *x*−1, *y*, *z*; (iv) −*x*, *y*+1/2, −*z*+3/2; (v) −*x*+1, *y*+1/2, −*z*+3/2.
